# Gastrodin improves neuroinflammation-induced cognitive dysfunction in rats by regulating NLRP3 inflammasome

**DOI:** 10.1186/s12871-022-01915-y

**Published:** 2022-12-01

**Authors:** Xue Zheng, Taowu Gong, Chunchun Tang, Yuanping Zhong, Lu Shi, Xu Fang, Dongqin Chen, Zhaoqiong Zhu

**Affiliations:** 1 Suzhou Medical College of Soochow University, Suzhou, 215000 Jiangsu Province China; 2 Department of Anesthesiology, Zunyi Maternal And Child Health Care Hospital, 287#, Zhonghua Road, Zunyi, 563000 Guizhou Province China; 3 Department of Anesthesiology, Affiliated Hospital of Zunyi Medical University, 149#, Dalian Road, Zunyi, 563000 Guizhou Province China

**Keywords:** Gastrodin, Neuroinflammation, NLRP3 inflammasome, Cognitive dysfunction, Network pharmacology, Molecular docking

## Abstract

Neuroinflammation is the main pathological mechanism of cognitive dysfunction caused by neurodegenerative diseases, and effective preventive and therapeutic measures are not available. We predicted the key targets of gastrodin’s effects upon neuroinflammation through *Network Pharmacology* and molecular docking. Then the predicted targets were used to study how gastrodin affected cognitive dysfunction triggered by lipopolysaccharide-induced neuroinflammation in rats and its mechanisms. Three-month-old male rats were intraperitoneally injected with lipopolysaccharide for 3 days (d), 7 d and 14 d respectively. Gastrodin improved learning and memory ability of rats with neuroinflammation. Lipopolysaccharide enhanced the levels of pro-inflammatory cytokines, such as TNF-α, IL-1β and IL-6, in rat hippocampus, which could be reversed by gastrodin. Gastrodin also inhibited the activation of microglia. Our findings suggested that gastrodin exerted neuroprotective effects in rats with neuroinflammation by impacting the TLR4-NF-kB-NLRP3 pathway. Therefore, gastrodin may be a potential therapeutic agent for neuroinflammation-induced cognitive dysfunction.

**Supplementary Information:**

The online version contains supplementary material available at 10.1186/s12871-022-01915-y.

## Introduction

As the global aging population continues to increase, cognitive impairment caused by neurodegenerative diseases has become a major problem affecting the quality of life of the elderly. Once cognitive dysfunction occurs, it will have a bad impact on the patient’s quality of life and work ability [1]. Since the occurrence and development of cognitive dysfunction is closely related to neuroinflammation mediated by inflammatory cytokines [2], neuroinflammation has gradually become a research hotspot [3, 4]. Microglia, the first line of defense in the brain, are mononuclear phagocytes of the central nervous system and can protect the brain from extracorporeal damage and the invasion of pathogens. They play a dominant role by providing cellular pseudopod with protrusions to maintain homeostasis in the nervous system [5, 6]. Activated microglia can release pro-inflammatory cytokines such as tumor necrosis factor-α (TNF-α) and interleukin-1β (IL-1β) and generate a large number of free radicals such as reactive oxygen species (ROS) to elicit an inflammatory response [7, 8]. Furthemore, neuroinflammation not only causes various structural and functional changes in the brain, but also leads to pathological activation of microglia and imbalance of cytokine regulation, thereby triggering neurological dysfunctions such as microglia reactivity and subsequent synapses function [9]. Previous studies have shown that the inflammatory response mediated by the activation of microglia plays an important role in the pathogenesis of cognitive dysfunction [10, 11]. Therefore, inhibiting microglia-mediated neuroinflammatory responses is a potential approach for the treatment of cognitive dysfunction.

*Gastrodia elata*, a traditional Chinese medicinal herb, is the tuber of the Orchidaceae Gastrodia. As the effective constituent of Gastrodia elata, gastrodin finds wide application clinically in anti-inflammation, anti-oxidation, hypoxia tolerance, sedation, alleviating pain, etc [12, 13]. Studies have shown that gastrodin can improve cognition by inhibiting the formation of amyloid beta (Aβ) and its fibrous plaques [14], suggesting that gastrodin may be a potential therapeutic drug for neuroinflammation concomitant with Alzheimer’s disease. Furthermore, gastrodin can inhibit the transcription cytokine NF-kB signaling pathway and phosphorylation of MAPK in lipopolysaccharide-stimulated microglia BV-2, thereby reducing neurotoxic pro-inflammatory mediators and pro-inflammatory cytokines [15]. Therefore, gastrodin could be a potential anti-inflammatory agent for treating neurodegenerative disease. However, the effect and mechanism of gastrodin on neuroinflammation-induced cognitive dysfunction in rats has not been reported yet.

Hence, we studied whether gastrodin can improve cognitive impairment in a rat model of lipopolysaccharide-induced neuroinflammation through *Network Pharmacology* and molecular docking to predict targets.

## Materials and methods

### *Network pharmacology* and molecular docking to predict targets

#### Screening of key targets

In order to find the action target of gastrodin, we used “gastrodin” and “neuroinflammation” as the key words, and the species was confined to “human”. Prediction was performed through Pubchem, PharmMapper, Genecards, and UniProt respectively to obtain the relevant targets.

#### Establishment of protein interaction network (PPI) and prediction of gene clusters and core genes

In order to explain the interaction between target proteins, common targets were entered into STRING for retrieval. The data of targets interaction network was obtained, and then imported into Cytoscape to obtain the protein network interaction map. Next, the map was imported into Cytoscape, and the MCODE module was used for gene cluster analysis and screening core targets.

#### GO functional enrichment analysis and KEGG pathway enrichment analysis

GO functional analysis and KEGG pathway enrichment analysis were performed on common targets using R language. GO functional analysis is mainly used to annotate gene functions, including biological processes (BP), cellular components (CC), and molecular functions (MF) [16, 17]. KEGG enrichment analysis facilitates enrichment analysis of common targets [18]. *P* < 0.05 was considered statistically significant in both GO and KEGG analysis.

#### Prediction of molecular docking

In order to further explore theoretically whether gastrodin was related to the key targets predicted by *Network Pharmacology*, computer-assisted molecular docking was used to predict them.

### Drugs and reagents

Gastrodin (CAS:62499–27-8) was purchased from Solarbio (Beijing, China). Lipopolysaccharide (#0000090043) was purchased from Sigma (USA). Antibodies for Iba-1 (ab283119) and NF-kB p65 (ab16502) were purchased from Abcam (USA). Antibodies for IL-18 (10663–1-AP), Caspase-1 (22915–1-AP), GAPDH (10494–1-AP) were purchased from Proteintech (USA). Antibodies for IL-1β (bs-6319R) and ASC (bs-6741R) were purchased from Bioss (Beijing, China). Antibodies for TLR4 (#35463) were purchased from SAB (Beijing, China). Antibodies for NLRP3(ET1610–93) were purchased from Huabio (Hangzhou, China). Rat TNF-α ELISA Kit (JL13202), Rat IL-1β ELISA Kit (JL20884), Rat IL-6 ELISA Kit (JL20896) were purchased from Jianglai (Shanghai, China).

### Animals, neuroinflammation model and grouping

Three-month old male SD rats were purchased from Laboratory Animal Center of Zunyi Medical University. All the animal experiments were approved by the Animal Experiment Ethics Committee of Zunyi Medical University (ethical review number: KLLY(A)-2019–089;Zunyi, China). The study was carried out in compliance with the ARRIVE guidelines. All the animals were treated according to internationally accepted principles. The rats were fed a standard diet and water in a controlled environment (the temperature was kept at 19–22 °C, the humidity was kept at 40–60%), under a 12 h dark/12 h light cycle.

Model establishment: a single intraperitoneal injection of lipopolysaccharide (5 mg/kg) was used to induce an animal model of neuroinflammation.

In the experiment, 3-month-old SD rats were randomly divided into 3d group (control group (CON), inflammation group (LPS), inflammation plus gastrodin group (LPS + GAS)), 7d group (CON, LPS, LPS + GAS), 14d group (CON, LPS, LPS + GAS). There were 8 rats in each group. Morris water maze test had been performed before the model was established. The test was performed again 3 d, 7 d and 14 d after intragastric administration of gastrodin. Based on the previous experimental results of the research group, the dose of gastrodin used in this experiment was 25 mg/kg.

### Morris water maze (MWM)

MWM is a cylindrical pool. The inner wall of the pool is artificially set with four quadrants, namely the first, second, third, and fourth quadrants, and a platform is placed in the center of any quadrant (the experimental platform is in the first quadrant, and the water depth is 2 cm higher than the platform). Some special shapes, such as triangles and circles, are placed above the midpoints of the four quadrants. During the experiment, the position of the markers remained unchanged to ensure that the rats could develop learning and memory. The water temperature was kept at 22 °C, and the experimental environment was kept quiet.

Mwm adaptive training includes place navigation text (PNT) and spatial probe test (SPT):

①PNT: Put the rat’s head toward the pool wall from the midpoint of the first, second, third, and fourth quadrants into the pool respectively. Then record the time since the rat enters the water to until it climbs onto platform with all its limbs, namely escape latency. Experiment rules: after the rat climbs onto the platform, if it stays for more than 2 seconds (s), it is regarded as qualified, otherwise it is regarded a failure to climb onto the platform; if it fails to climb onto the platform within 120 s after entering the water, the acquisition system automatically turns off, and the escape latency is recorded as 120 s; guide the rat to the platform where it stays for more than 5 s to help the rat learn and memorize the location of the platform.

②SPT: After 5 d of continuous PNT, the platform is removed the following day. Then choose a location to put the rat into the water (the midpoint of the fourth quadrant was chosen as the entry point for the space exploration experiment in this experiment). In the following 120 s, record how many times the rat crosses the platform, how many times it crosses the platform quadrant, the percentage of crossing the platform and the percentage of travelling distance.

### Elisa

The hippocampal tissues of the rats in each group were collected 3d, 7d and 14d after neuroinflammation had been modelled. Then the tissues were placed in a pre-cooled glass grinder and a certain amount of PBS (pH 7.4) was added. Next, the hippocampus was fully homogenized by hand. After being centrifuged for at 2000–3000 round/minute to rpm, supernatant was collected for detection. The standard wells and the sample wells to be tested were loaded respectively, which was followed by addition of enzymes, incubation, washing, coloration and terminating reaction. Finally, OD value was read at 450 nm by a microplate reader.

### Hematoxylin and eosin (HE) staining

The whole brains of rats were taken at each time point, fixed in 4% paraformaldehyde, and then embedded in paraffin for later use. The tissues were cut into 5-μm-thick sections, placed in xylene for dewaxing and hydrated with gradient ethanol. Then, they were with hematoxylin for 5 min, differentiated by alcohol and stained with eosin solution for 2 min. Finally, they were dehydrated and sealed with gum. The slice was observed under electron microscope for cellular morphology.

### Immunofluorescence

The whole brains of rats were taken at each time point, fixed in 4% paraformaldehyde, and then embedded in paraffin for later use. The tissues were cut into 5-μm-thick slices. Then, they were placed into xylene and ethanol for dewaxing. After being placed into citric acid for antigen retrieval, they were blocked with BSA for 30 min at room temperature. Slices were incubated with anti-Iba-1 overnight. Next, they were washed with PBS and incubated with secondary antibody for 2 hours (h). The nuclei had been stained with DAPI for 5 min in the dark, the slices were covered with glass slides. Samples were observed with a confocal microscope and analyzed using ipWin32.

### Western blot

After the hippocampus had been completely lysed, the supernatant was collected by centrifugation. After protein quantification by BCA method, protein separation was carried out in the electrophoresis device by polyacrylamide gel electrophoresis. Then, the proteins were transferred to PVDF membrane, blocked with 5% nonfat milk powder, and mixed with corresponding primary antibodies (Iba-1, NLRP3, ASC, Caspase-1, TLR4, NF-kBp65, IL-1β, IL-18) overnight. After that, the proteins were incubated with secondary antibody. Immunoreactive bands were detected using a chemiluminescent substrate and images were acquired using an exposure meter.

### Statistical analyses

All data are shown as the mean ± standard error of the mean (x ± SEM). Statistical data were processed using IBM SPSS Statistics for Windows, version 18.0 and GraphPad Prism 6. One-way analysis of variance was used to determine the least significant difference in each group (homogeneity of variance and conformation to normal distribution). Otherwise, Tamhane T2 test was used to determine the significant differences between groups. Differences were considered statistically significant at *P* < 0.05.

## Results

### Predicting targets by *network pharmacology* and molecular docking

#### Screening key targets

**Fig. 1:**
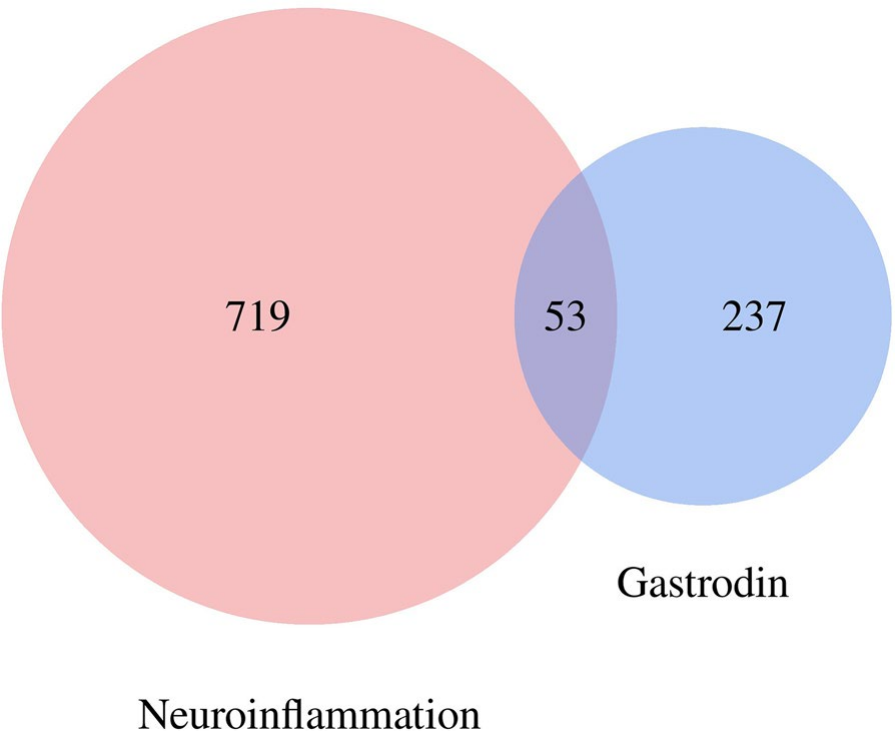
Venn diagram was applied to presenting all targets of Gastrodin and Neuroinflammation

Set “gastrodin” and “neuroinflammation” as keywords. 290 targets of gastrodin and 772 targets of neuroinflammation were predicted through Pubchem, PharmMapper, Genecards, and UniProt. 53 common drug-disease targets were obtained after taking intersection (Fig. 1).

#### PPI network, gene clusters and core genes

**Fig. 2:**
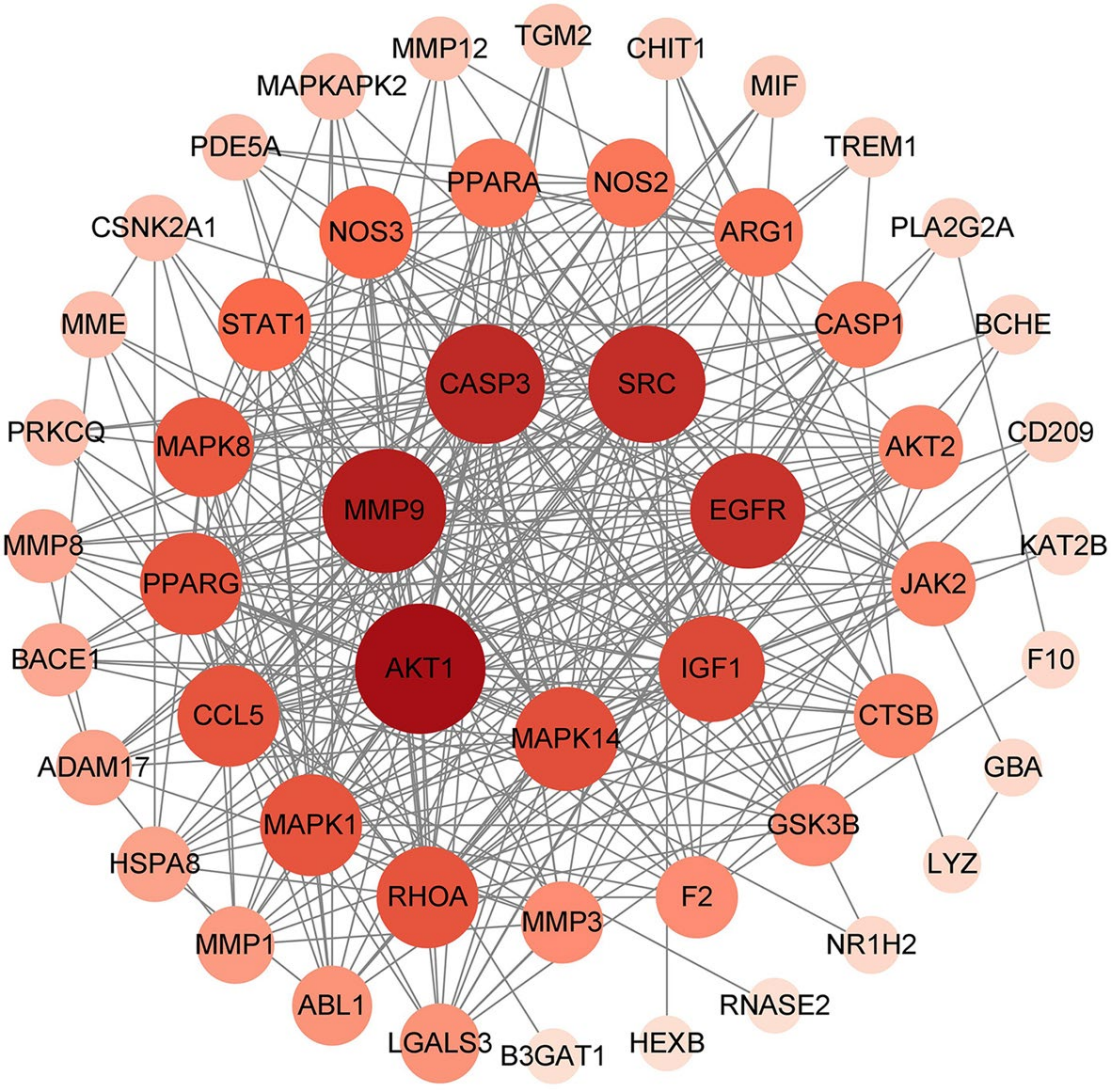
PPI network diagram of core targets

**Fig. 3:**
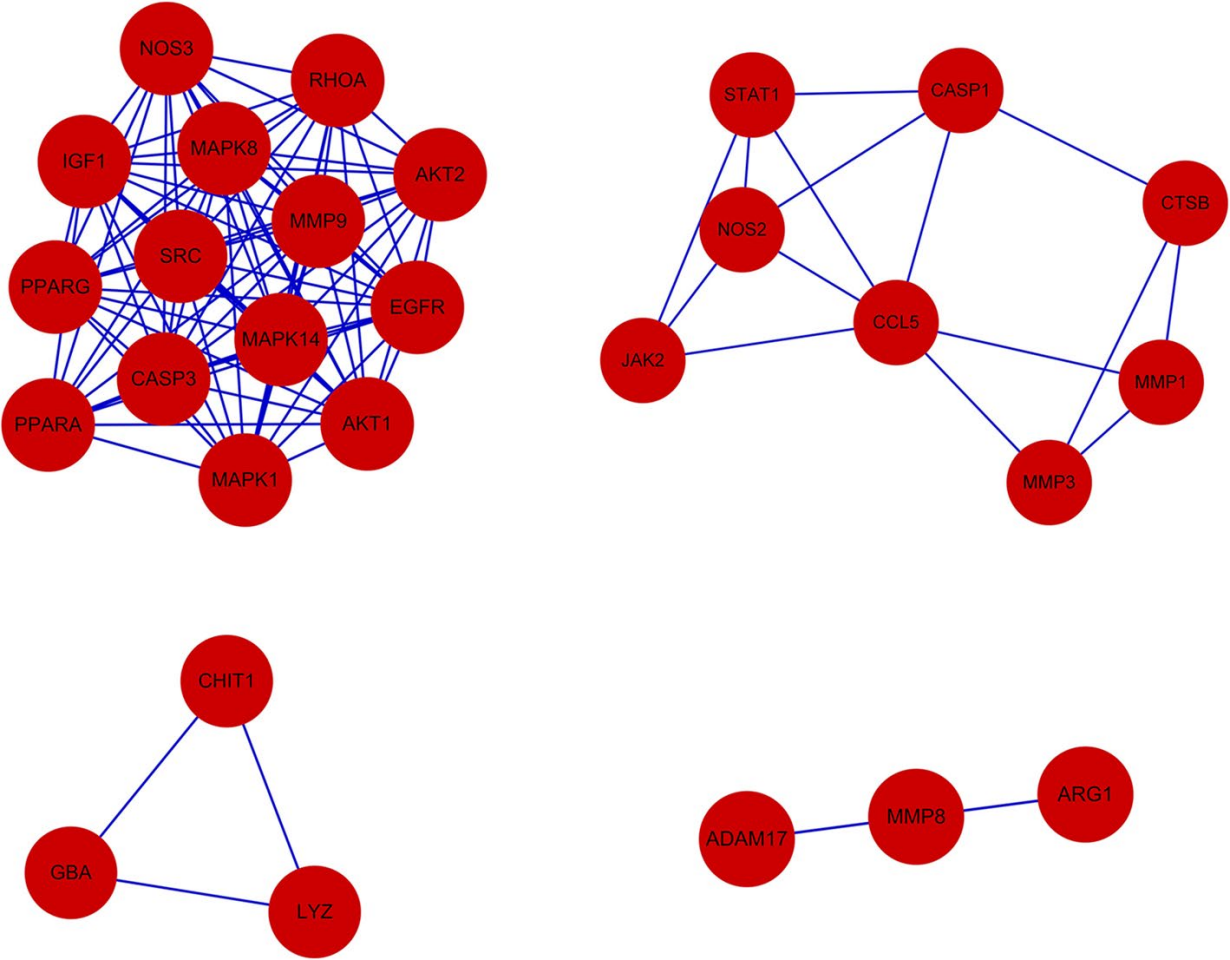
Gene cluster analysis based on MCODE module

The above 53 common targets were imported into the STRING database for retrieval to obtain the network relationship data of targets interactions. Then, the data were imported into Cytoscape to obtain the protein interaction network diagram (Fig. 2). The complete PPI network map was imported into Cytoscape. MCODE module was used for gene cluster analysis and core target screening. A total of 4 gene clusters (Fig. 3) and 3 core genes were obtained. The core genes are IGF1, CASP1, GBA.

#### GO functional enrichment analysis and KEGG pathway enrichment analysis

**Fig. 4:**
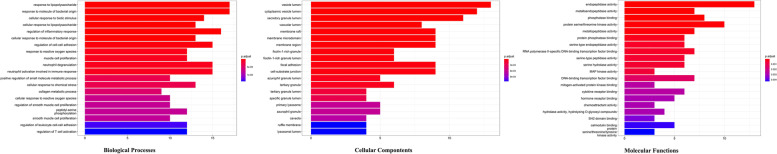
Gene Ontology functional enrichment analysis

GO functional analysis and KEGG pathway enrichment analysis were performed on common targets using R language. After running the 53 common targets through the R language, three parts, BP, CC and MF, were selected for GO analysis. The results showed that the sets of intersecting genes were enriched in 1237 BP pathways; the intersecting genes were enriched in expression process of 33 CCs; the intersecting genes were enriched in 81 processes related to MF (Fig. 4).

**Fig. 5:**
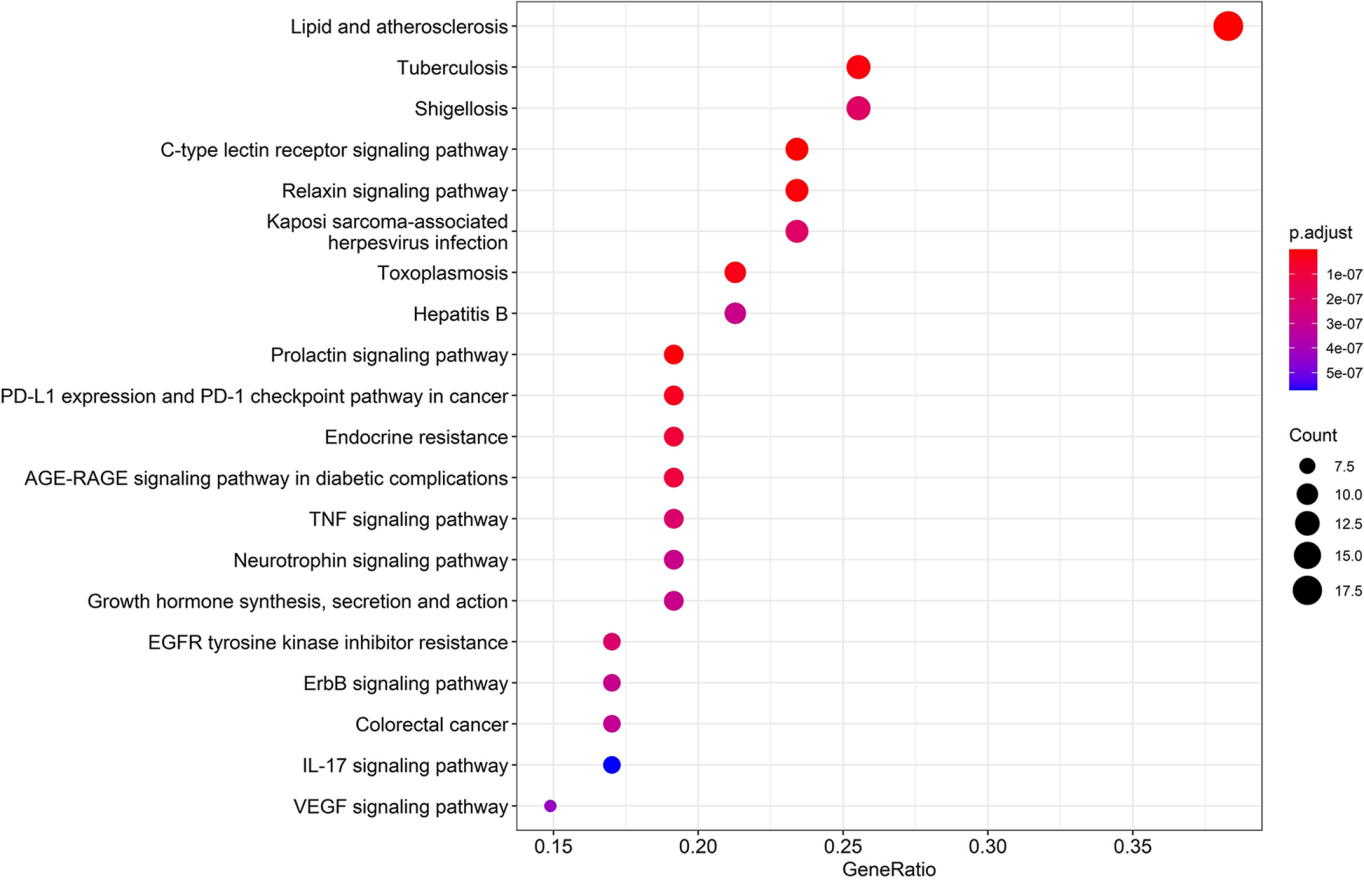
Kyoto Encyclopedia of Genes and Genomes pathway enrichment analys [19–21]_._

**Fig. 6:**
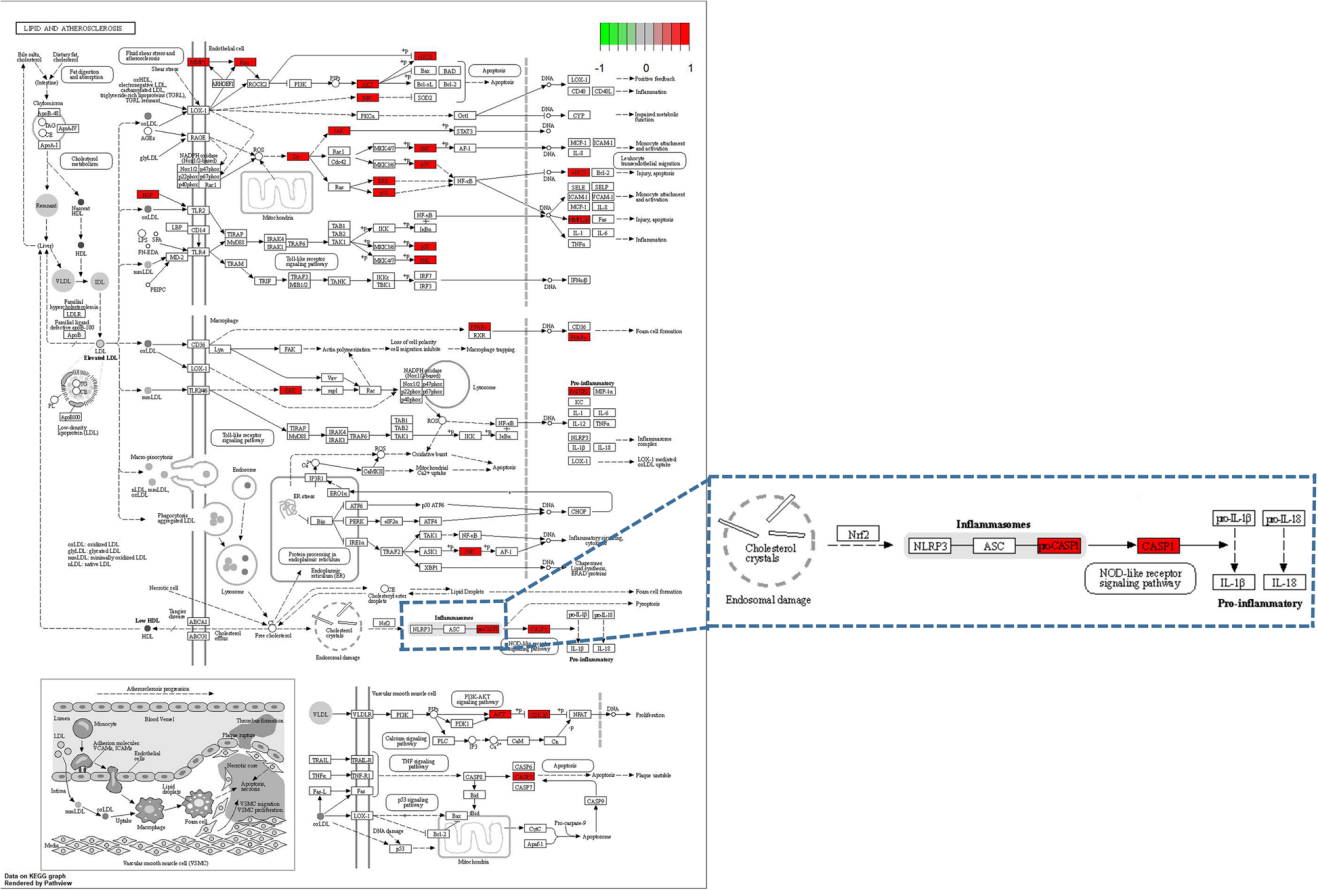
Lipid and atherosclerosis pathway [19–21]_._

In order to study the potential pathways of gastrodin in neuroinflammation, we carried out KEGG enrichment pathway analysis and obtained a total of 136 KEGG pathways (Fig. 5). Among them the Lipid and atherosclerosis pathways have the greatest weight. This pathway includes the core gene CASP1, which is one of the components of the NLRP3 inflammasome (Fig. 6).

#### Molecular docking prediction of gastrodin and NLRP3

**Fig. 7:**
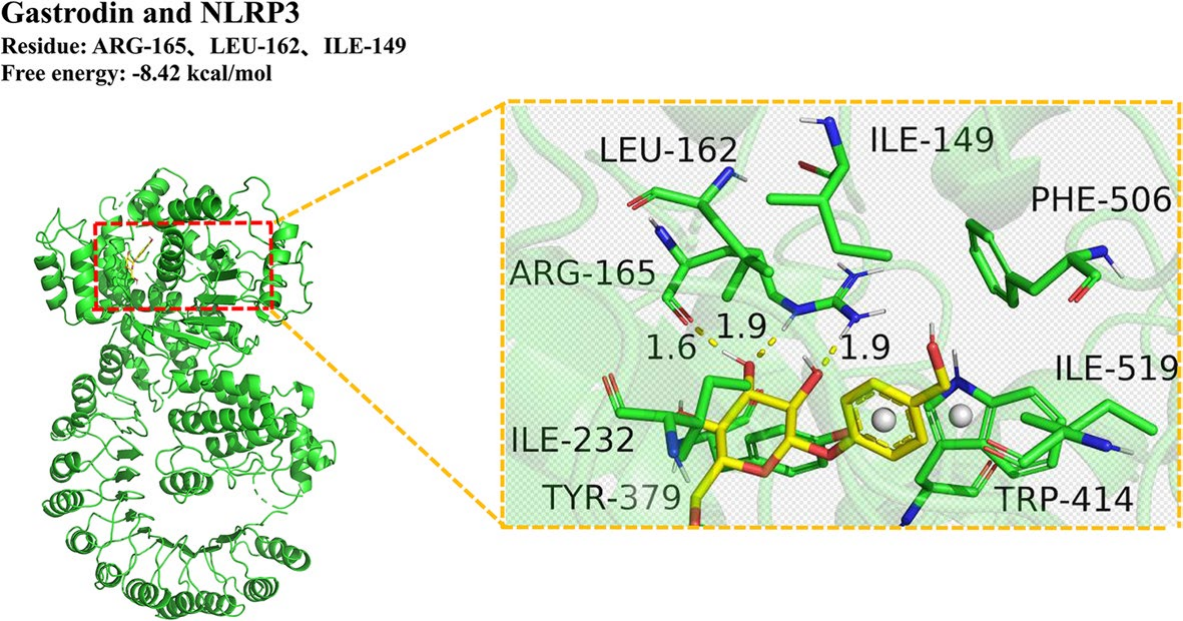
Gastrodin and NLRP3 molecular docking

Computer-aided molecular docking was used to predict gastrodin and NLRP3. A negative score of the docking result indicates that the compound could actively bind to the target protein. The absolute value of the score was in proportion to the binding ability. Gastrodin could form hydrogen bond interactions with arginine, leucine and isoleucine of NLRP3 respectively. These existing interactions could enhance the stability of gastrodin in the NLRP3 protein pocket (Fig. 7).

### Gastrodin improves cognitive function in rats with neuroinflammation

**Fig. 8:**
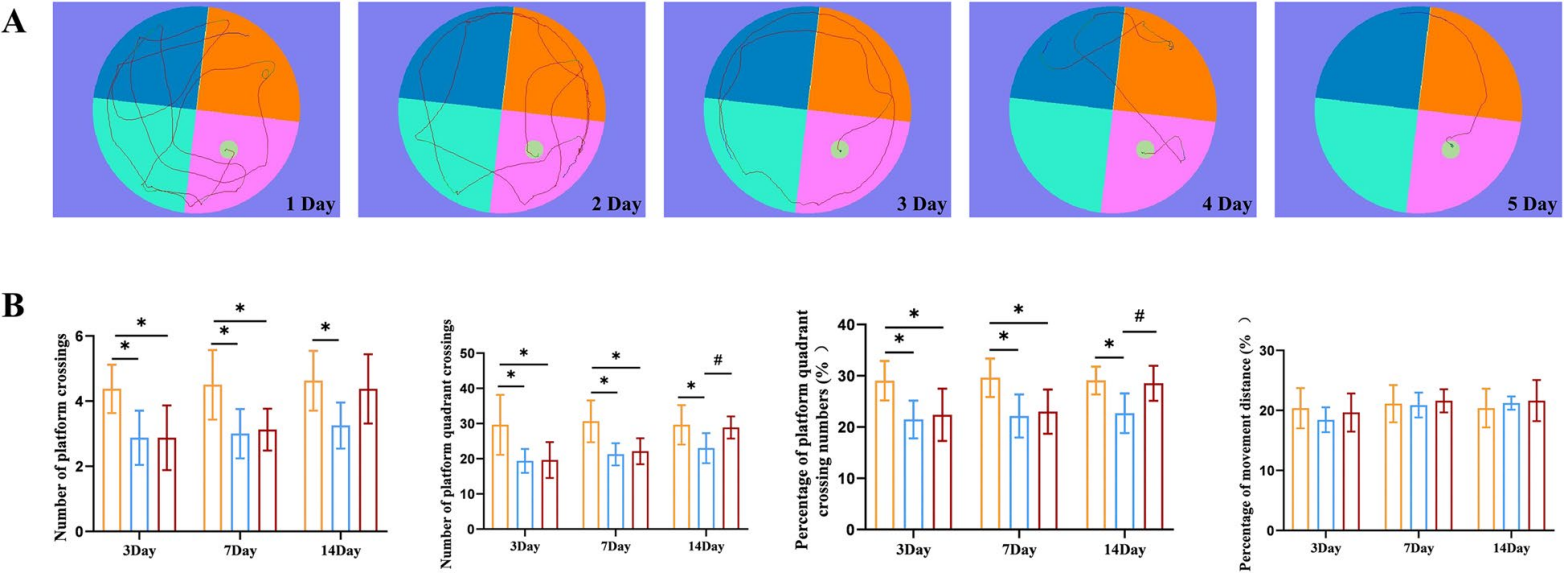
Morris water maze test. **a** Representative image of the escape latency in Morris water maze (training phase for 5 consecutive days). **b** Effects of GAS on learning and memory in rats based on number of platform crossings, number of platform quadrant crossings, percentage of platform crossing numbers and percentage of movement distance. Results are the mean ± SD. (*n* = 8). ^*^P < 0.05 compared with CON rars, ^#^*P* < 0.05 compared with LPS rats

Before modeling, MWM test was used to conduct PNT on rats for 5 d. As training days passed by, the rats gradually developed learning and memory, formed good learning and memory on the 4th day, and had stable learning and memory on the 5th day (Fig. 8a). Next, we explored the effect of gastrodin on cognitive function in rats with neuroinflammation. MWM test was performed by gavage of gastrodin for 3 d, 7 d and 14 d after modeling. There was no significant difference based on the results of the percentage of travelling distance of the rats in each group. Compared with the CON group, there was decrease in how many times the rats had crossed the platform quadrants and the percentage of crossing platform quadrants in the LPS group. After GAS processing, there was significant increase in the times for crossing the platform quadrants and the percentage of crossing platform quadrants (Fig. 8b).

### Gastrodin reduces pro-inflammatory cytokines

**Fig. 9:**
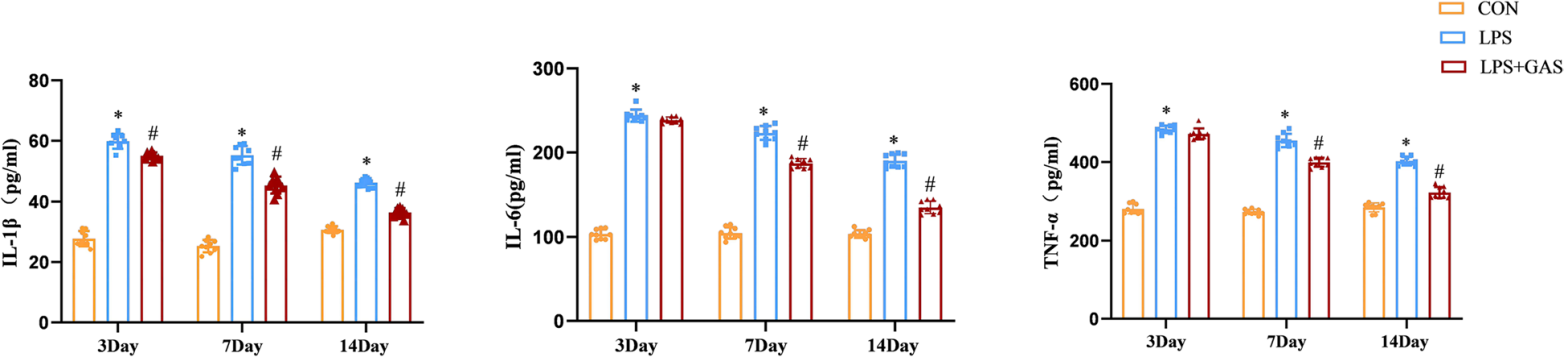
Effect of Gastrodin on cytokine production in rats hippocampus after neuroinflammation. **a** IL-β level in supernatant of rats’ hippocampus. **b** IL-6 level in supernatant of rats’ hippocampus. **c** TNF-α level in supernatant of rats’ hippocampus. Results are the mean ± SD. (n = 8). ^*^P < 0.05 compared with CON rars, ^#^*P* < 0.05 compared with LPS rats

Given the critical role of neuroinflammation in cognitive dysfunction, we next examined the effect of gastrodin on inflammatory cytokines in the rats’ hippocampus by ELISA. Compared with the CON group, the inflammatory cytokines IL-1β, IL-6 and TNF-α increased in the LPS group. The inflammatory cytokines decreased after gastrodin gavage, indicating that gastrodin had a strong inhibitory effect on the hippocampal inflammatory response in rats (Fig. 9).

### Gastrodin improves damaged hippocampal neuronal cells

**Fig. 10:**
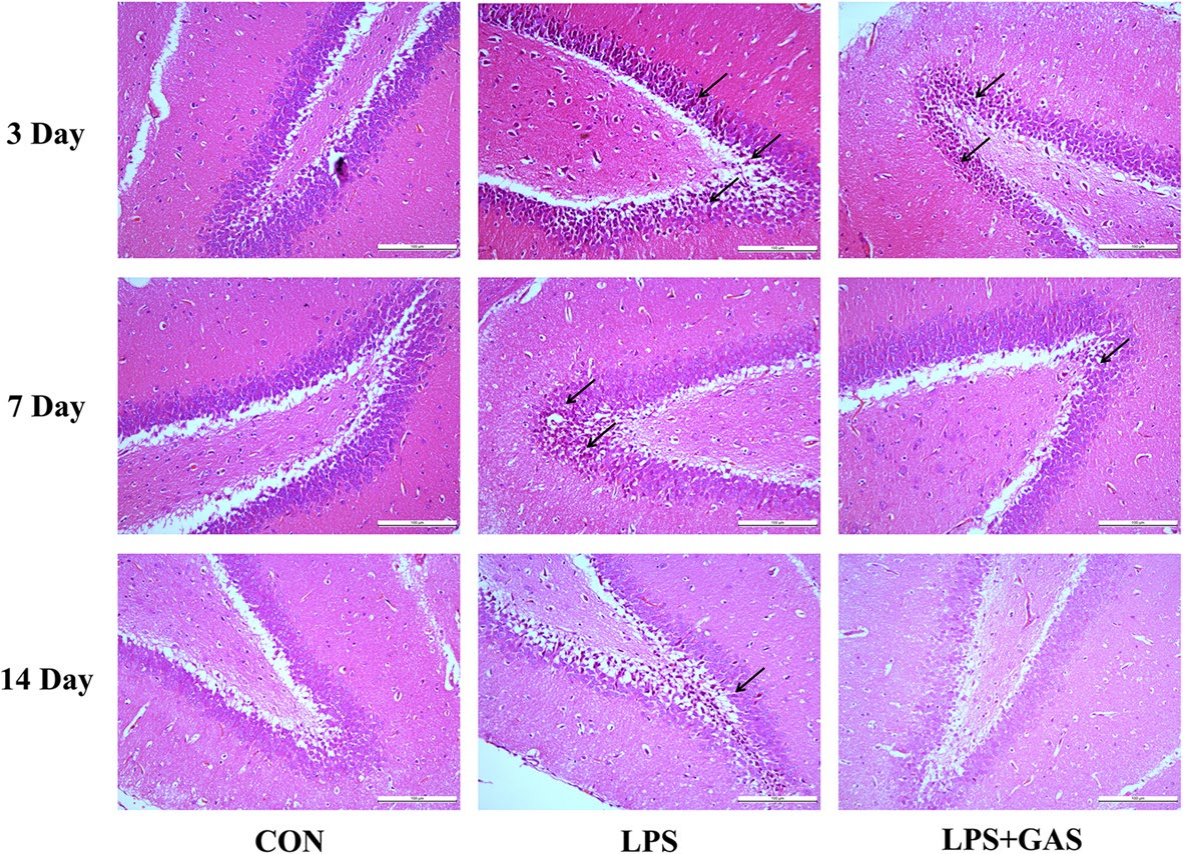
HE staining of neuronal cells in hippocampus. CON groups were no cell edema, necrosis, nuclear pyknosis, and hyperchromasia. Neurons in the LPS groups suffered necrosis, pyknosis and hyperchromatism to different extent. LPS + GAS groups had less neuronal cell necrotic, pyknotic and hyperchromatic

HE staining was used to observe how gastrodin ameliorated hippocampal neurons in rats with neuroinflammation in terms of morphology. The size and shape of hippocampal neurons in the 3d, 7d, and 14d CON groups were normal. There was no cell edema, necrosis, nuclear pyknosis, and hyperchromasia. In 3d LPS group, many neuron cells suffered necrosis; the cellular volume became smaller; the cytoplasm was stained red and the nuclei were pyknotic and hyperchromatic. As days passed by, the necrotic foci of neuronal cells shrank and the volume gradually expanded in the 7d and 14d groups. Compared with the LPS group, the LPS + GAS group had less neuronal cell necrosis, less nuclear pyknosis and hyperchromasia (Fig. 10).

### Gastrodin inhibits the activation of microglia in rats’ neuroinflamed hippocampus

**Fig. 11:**
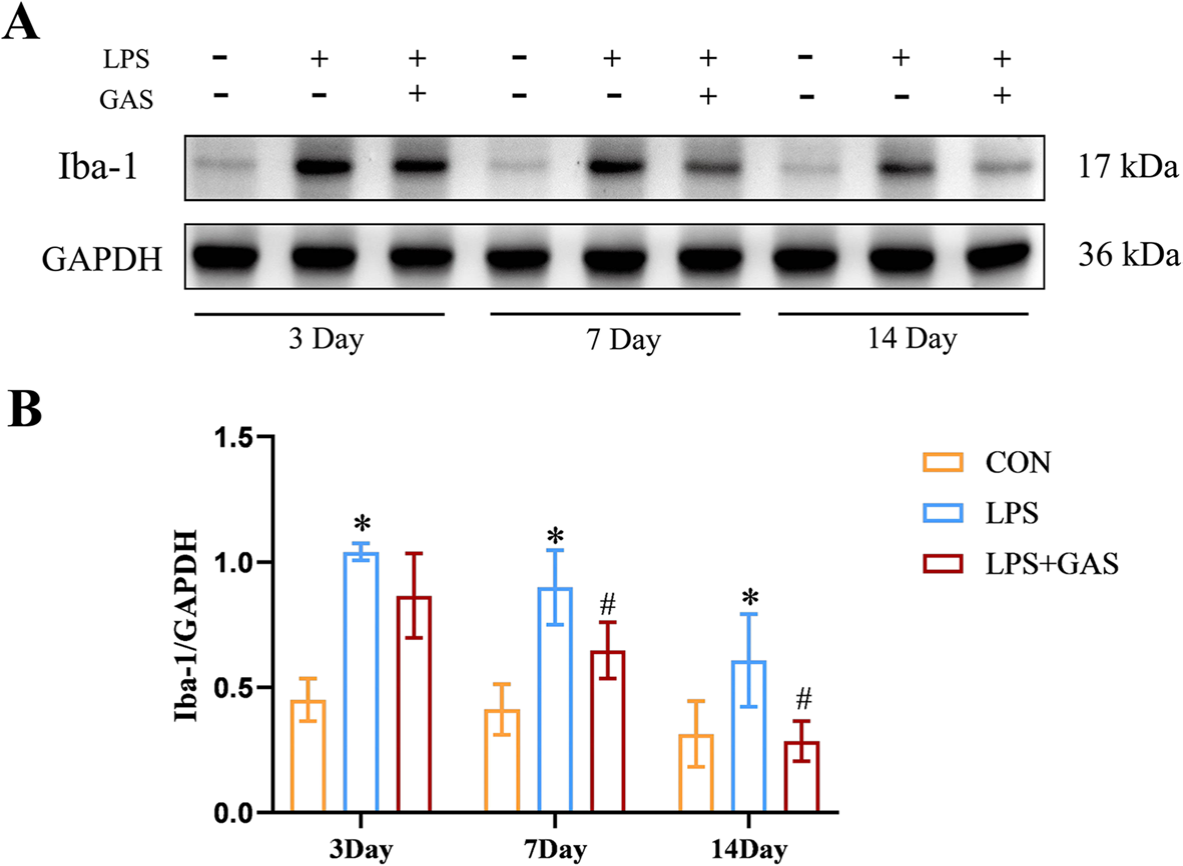
Effects of GAS on microglia activation in rats’ hippocampus. a Western blot assay of Iba-1 in rats’ hippocampus. b Related to (a), quantiative analysis of Iba-1 protein expression level. Results are the mean ± SD. (*n* = 3). ^*^P < 0.05 compared with CON rars, ^#^*P* < 0.05 compared with LPS rats

**Fig. 12:**
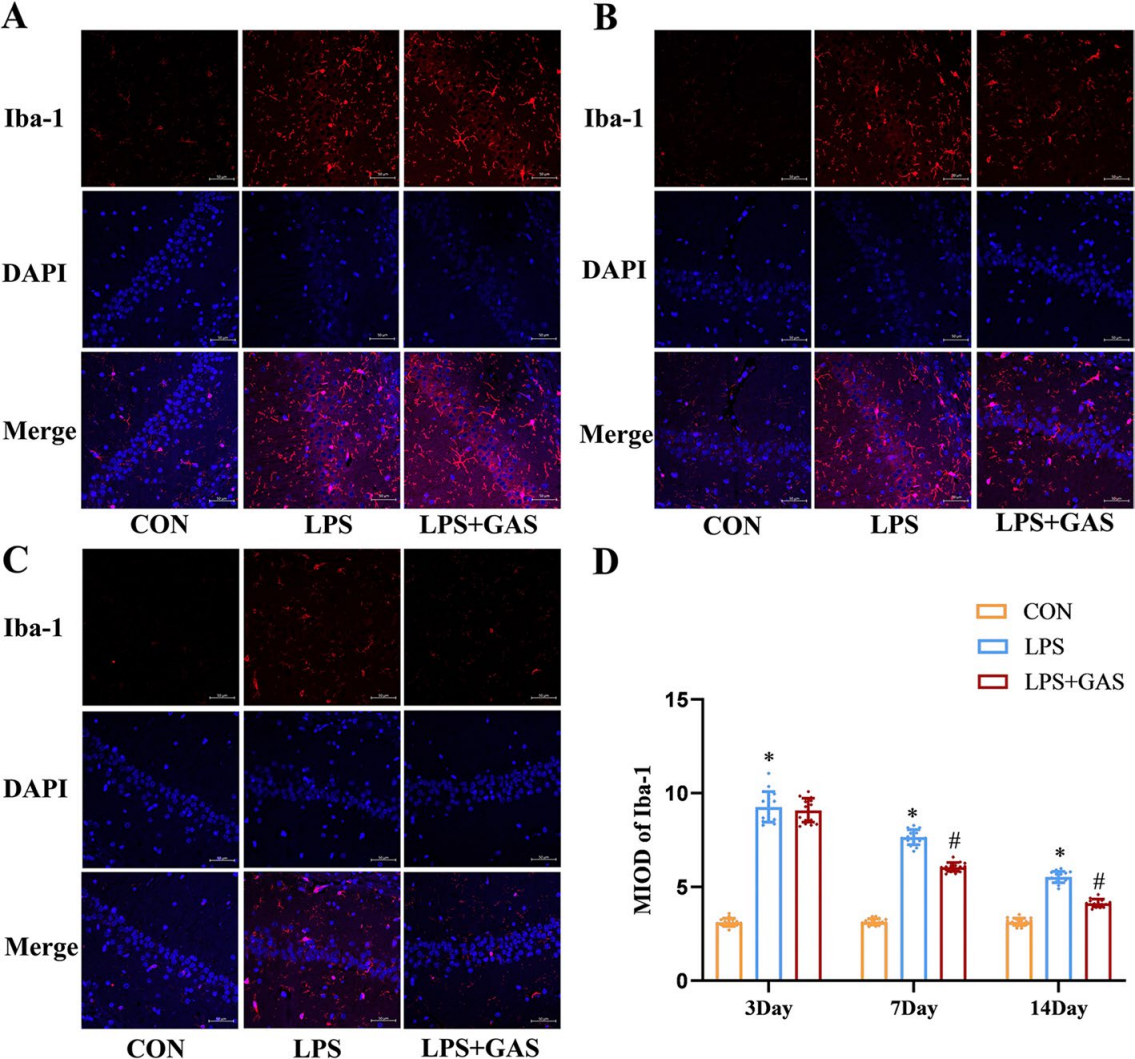
Effects of GAS on microglia activation in rats’ hippocampus after neuroinflammation. (a), (b) and (c) Immunofluorescence results of Iba-1 of treated rats’ hippocampus in 3, 7, 14 days. d Related to (a), (b), (c), mean IOD of immunofluorescence pictures of Iba-1 in hippocampus. Results are the mean ± SD. (*n* = 4). ^*^P < 0.05 compared with CON rars, ^#^*P* < 0.05 compared with LPS rats

The results of Western blot (Fig. 11) and immunofluorescence (Fig. 12) showed that the expression of Iba1 in the CON group was very low. The expression of Iba1 in the 3d LPS group was the highest. However, the expression gradually decreased as days went by. The expression of the LPS + GAS group was reduced compared with the LPS group.

### Gastrodin inhibits the NLRP3 inflammasome through the TLR4-NF-κB-NLRP3 pathway

**Fig. 13:**
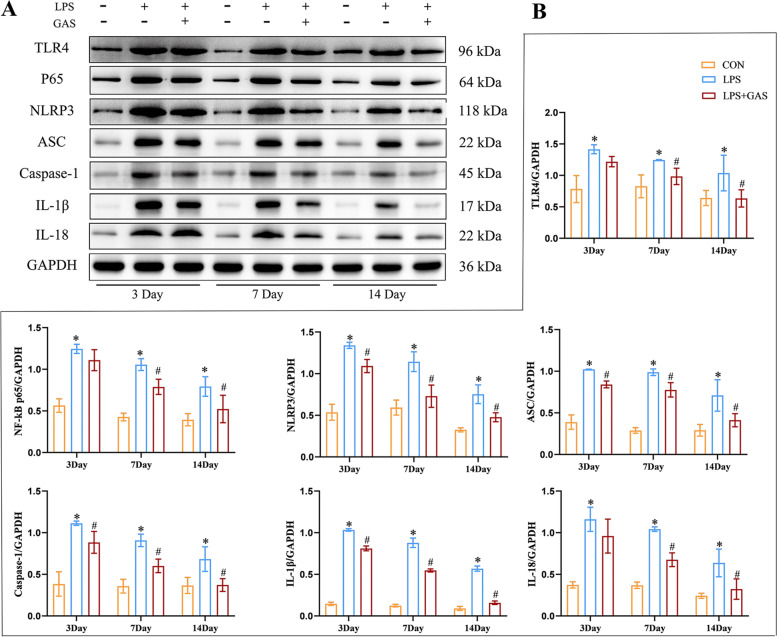
Western blot assay of TLR4-NF-kB-NLRP3 pathway of rats’ hippocampus. (A) Western blot assay of TLR4, NF-kB, NLRP3, ASC, Caspase-1, IL-1β, IL-18 protein. (B) Related to (A), quantitive analysis of proteins expression level. Results are the mean ± SD. (n = 3). ^*^P < 0.05 compared with CON rars, ^#^*P* < 0.05 compared with LPS rats

The results of our experiments showed (Fig. 13) that compared with the CON group the expressions of TLR4, NF-κB (P65), NLRP3, ASC, Caspase-1 and the downstream inflammatory cytokines IL-1β and IL-18 increased in the LPS group. LPS + GAS group histone expression decreased after gastrodin treatment.

## Discussion

As the aging process of the world’s population accelerates, more and more neurodegenerative diseases, such as Alzheimer’s and Parkinson’s diseases, are being diagnosed. Cognitive dysfunction is mainly manifested as impaired learning and memory ability, which seriously affects patients’ life quality and puts heavy economic burden on families and the society. There are many theories on its pathogenesis, such as neuroinflammation, mitochondrial damage, elevated Aβ and phosphorylated Tau protein, etc., which can partially explain the pathogenesis [22–25]. However, a relatively acceptable view is that neuroinflammation is one of the main causes at present [26–28]. Microglia, as neuropathological sensors [29], are widely considered to be beneficial or detrimental, especially in neurodegenerative diseases. Activated microglia primarily remove necrotic cells and tissues in neuroinflammatory responses. Nevertheless, uncontrolled neuroinflammation mediated by microglia may promote the occurrence and development of neurodegenerative diseases [30]. To ameliorate neurodegenerative diseases caused by microglia-mediated neuroinflammation, we identified gastrodin as a potential drug. It has been reported that in addition to wide application in anti-convulsion, alleviating pain, and sedation [31], gastrodin can also be anti-oxidative, anti-inflammatory, anti-epileptic, anti-obesity, anti-anxiety, and improve learning and memory [32–37]. Given that LPS has been widely used to modal mutiple cellular and animal inflammation [38] and the anti-inflammatory effect of gastrodin, we have studied that gastrodin can improve neuroinflammation in LPS-induced rats, suggesting that gastrodin has a protective effect on learning and memory in cognitive dysfunction. This finding is consistent with that of Wang et al. [39]. When tissues or organs are severely stimulated, inflammatory cells will release a large number of inflammatory mediators, thus aggravating the occurrence of inflammatory responses [40]. Therefore, we detected the expressions of pro-inflammatory cytokines IL-1β, IL-6, and TNF-α in rats’ hippocampus by ELISA to evaluate the severity of neuroinflammation. The results showed that gastrodin reduced the release of pro-inflammatory cytokines in the hippocampus of neuroinflamed rats.

Next, we explored the underlying mechanisms of how gastrodin ameliorated neuroinflammation. Network pharmacology prediction was performed by entering the keywords “gastrodin” and “neuroinflammation” in the database. Then, the screened targets underwent molecular docking. The prediction results showed that a total of 53 common targets and 136 KEGG pathways were obtained. Next, three core targets and the pathway with the most weight (Lipid and atherosclerosis) were integrated. Finally the core target to be verified was CASP1, which is one of the components of the NLRP3 inflammasome. Afterwards, we conducted molecular docking of gastrodin with NLRP3. The results showed that the docking score is − 8.42 kcal/mol. Gastrodin could form hydrogen bond and interact with the amino acid active groups of ARG-165, LEU-162, and ILE-149 of NLRP3, respectively, indicating that gastrodin can independently actively bind to NLRP3 protein and may take effect.

The occurrence of inflammation may be accompanied by overactivation of the inflammasome, and the NLRP3 inflammasome is one of the hot topics in inflammation research in recent years [41, 42]. The NLRP3 inflammasome is a multi-protein complex composed of intracellular pattern recognition receptor NLRP3 protein, adaptor protein ASC and protein cleavage enzyme caspase-1 [43]. Classical NLRP3 inflammasome activation is accomplished in two steps under dual signaling (inflammasome priming and inflammasome activation) [44]. At present, more and more studies on neurological diseases have found that abnormal activation of NLRP3 inflammasome is involved in the occurrence and progression of diseases, and is mainly expressed in microglia and neurons [45–49]. Hyperactivated NLRP3 inflammasome and IL-1β-mediated inflammatory responses in microglia are involved in hypoxemia and isoflurane-induced cognitive impairment in adult rats [50, 51]. In this study, we found that the cognitive dysfunction caused by LPS-induced neuroinflammation in rats was manifested by increasing protein expression of the NLRP3 inflammasome complex-NLRP3, ASC, and Caspase-1 while the protein expression decreased after gastrodin treatment. It indicated that gastrodin could regulate the NLRP3 inflammasome to improve cognitive function.

In the central nervous system, microglia become hyperactive in the immune response to brain injury and could boost the body’s defense mechanisms by destroying invading pathogens. Activated microglia affect peripheral astrocytes and neurons, thereby promoting tissue repair. However, chronic hyperactivation of microglia produces excessive inflammatory mediators and neuronal damage. In the present study, the protein expression of the microglial marker Iba-1 increased in the LPS-mediated inflammatory response. The immunofluorescence results indicated a significant increase in microglial activation. These changes were reversed after gastrodin treatment. Meanwhile, the results of HE staining showed that the hippocampal neurons in the LPS group had extensive necrosis, smaller cellular volume, red-stained cytoplasm, nuclear pyknosis, and hyperchromasia. In the LPS + GAS group, the neuronal cellular necrosis obviously reduced and the pyknosis and hyperchromasia improved after gastrodin treatment.

Accumulating evidence suggests that TLR4 and its downstream NLRP3 signaling pathway play an important role in the pathogenesis of microglia-mediated neuroinflammation [52]. TLR4 can generate excessive inflammatory mediators and cytokines after binding to LPS endotoxin, thereby activating a series of responses. Therefore, inhibiting the binding of LPS to TLR4 is an interesting therapeutic approach to treat neuroinflammatory diseases.

In this study, we provide evidence of involvement in downstream inflammatory responses by regulating the TLR4-NF-κB-NLRP3 pathway. Based on the study, it was found that LPS caused the overexpression of TLR4 and NLRP3 inflammasomes possibly by activating NF-κBp65, thereby upregulating Iba-1 inflammatory mediators. In addition, the expression levels of NLRP3, Caspase-1, ASC, IL-1β, and IL-6 proteins are closely related to the activation of the NLRP3 inflammasome [53]. It needs to be noted that gastrodin treatment can inhibit increase in the expression of TLR4, NF-κBp65, and IL-1β and IL-18 downstream of NLRP3. These findings support the view that regulation of the TLR4-NF-κB-NLRP3 pathway is critical for protection of microglia by gastrodin and inhibition of the NLRP3 inflammasome. Therefore, gastrodin may be a safe and effective drug for preventing neuroinflammation-induced cognitive dysfunction.

## Conclusion

This study shows that gastrodin can attenuate the activation of the NLRP3 inflammasome for the treatment of cognitive dysfunction caused by neuroinflammation in rats.

## Supplementary Information

**Additional file 1.** Original image of Western Blot.

## Data Availability

All data presented in this study are available from corresponding author on reasonable request.
