# Supplementary material for: Gastrodin improves neuroinflammation-induced cognitive dysfunction in rats by regulating NLRP3 inflammasome
Source: BMC Anesthesiol. 2022 Dec 1;22:371. doi: 10.1186/s12871-022-01915-y (PMC9714247; doi:10.1186/s12871-022-01915-y)

**Additional file 1: Original image of Western Blot**

**Fig. 11**

Iba-1:


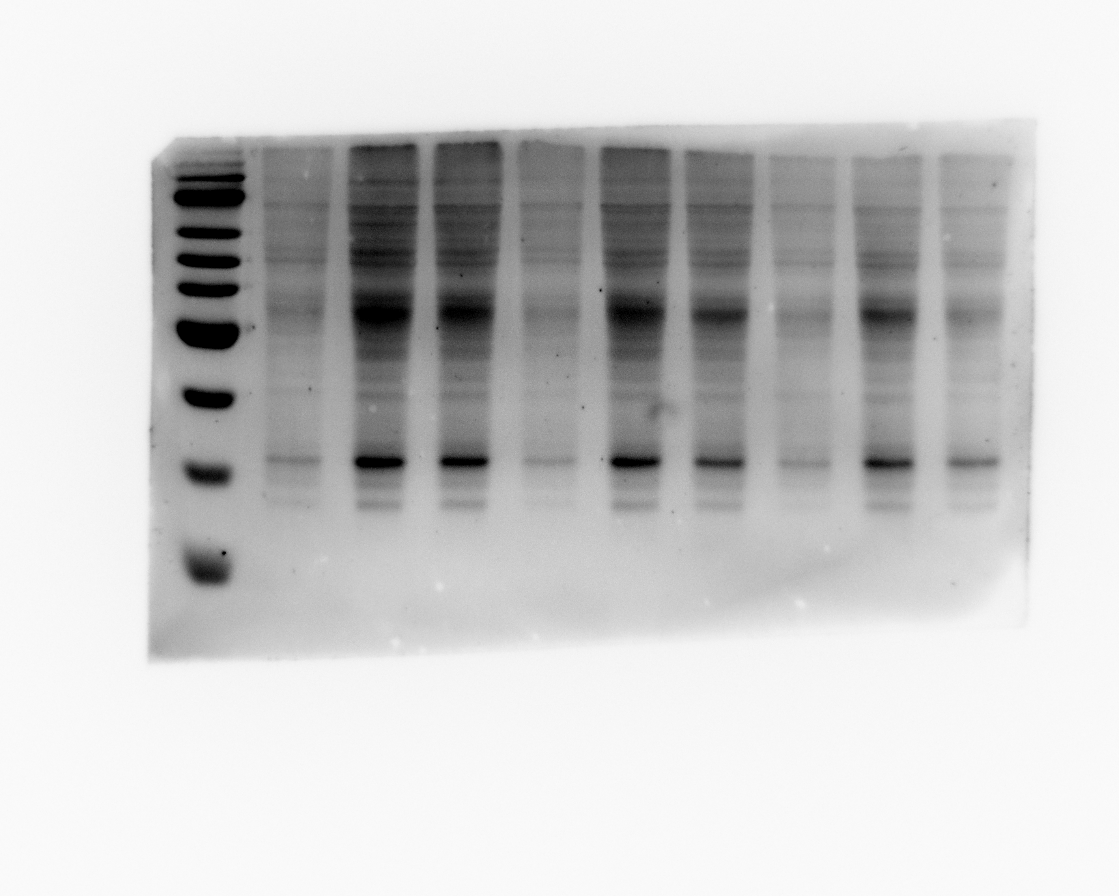


GAPDH:


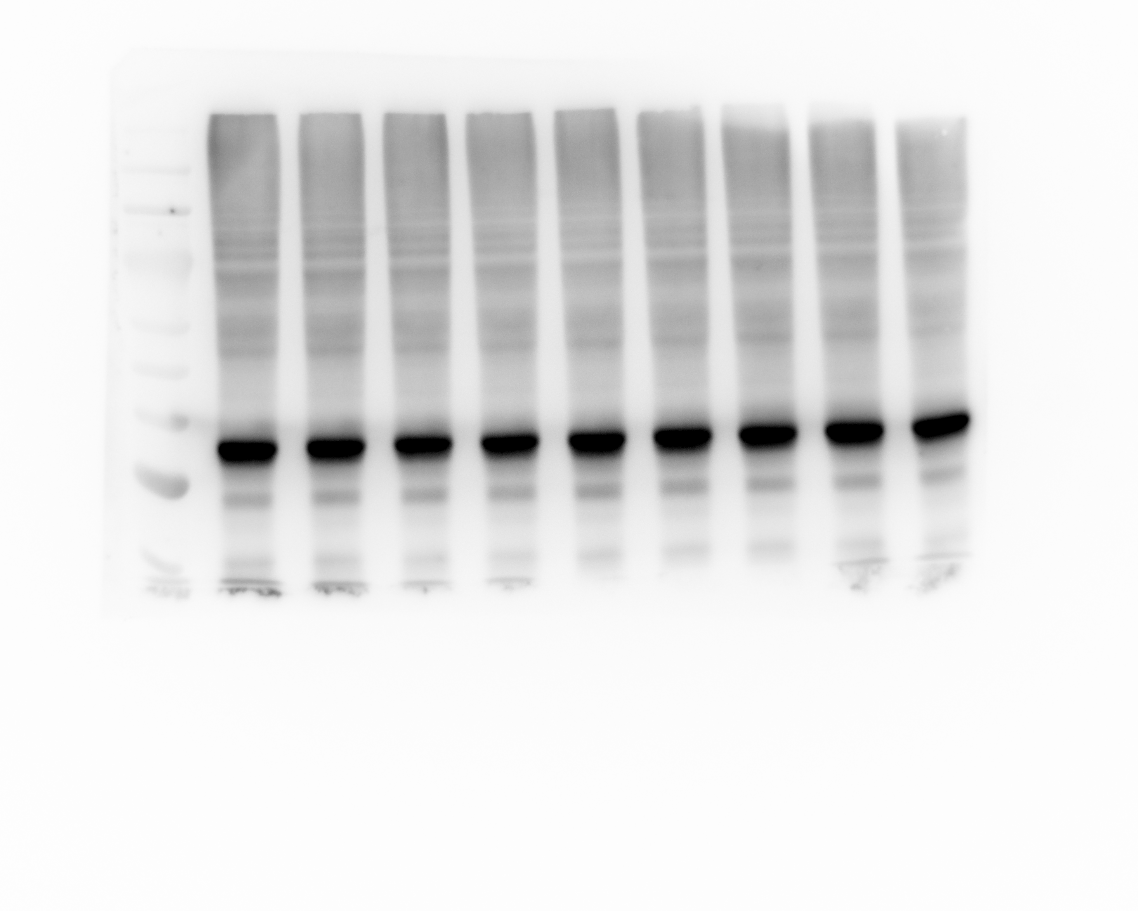


**Fig. 13**

TLR4:


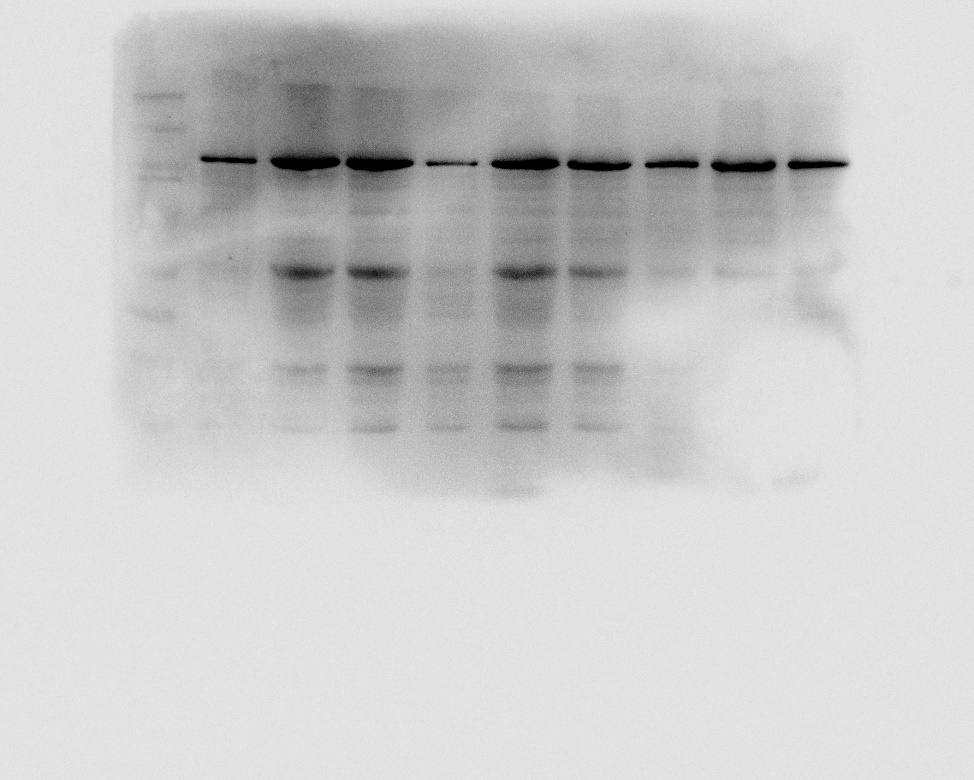


P65:


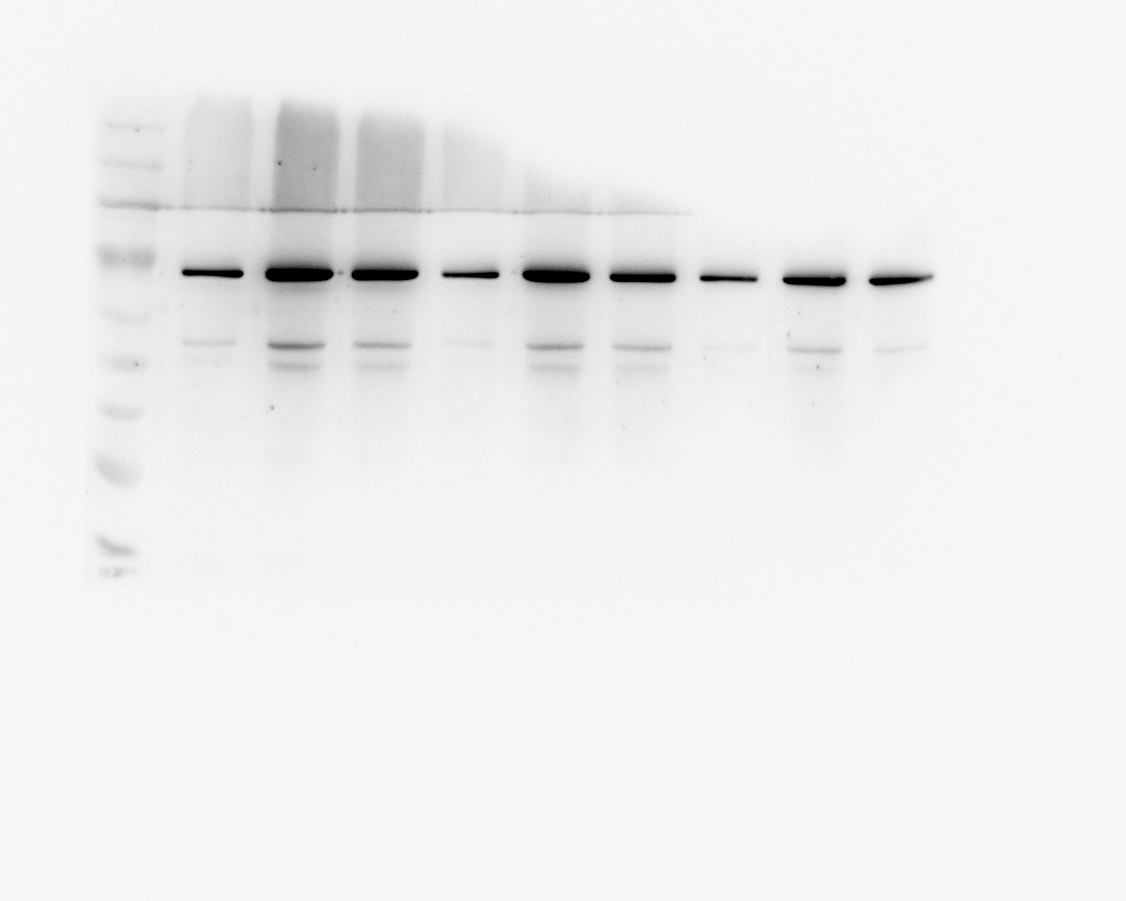


NLRP3:


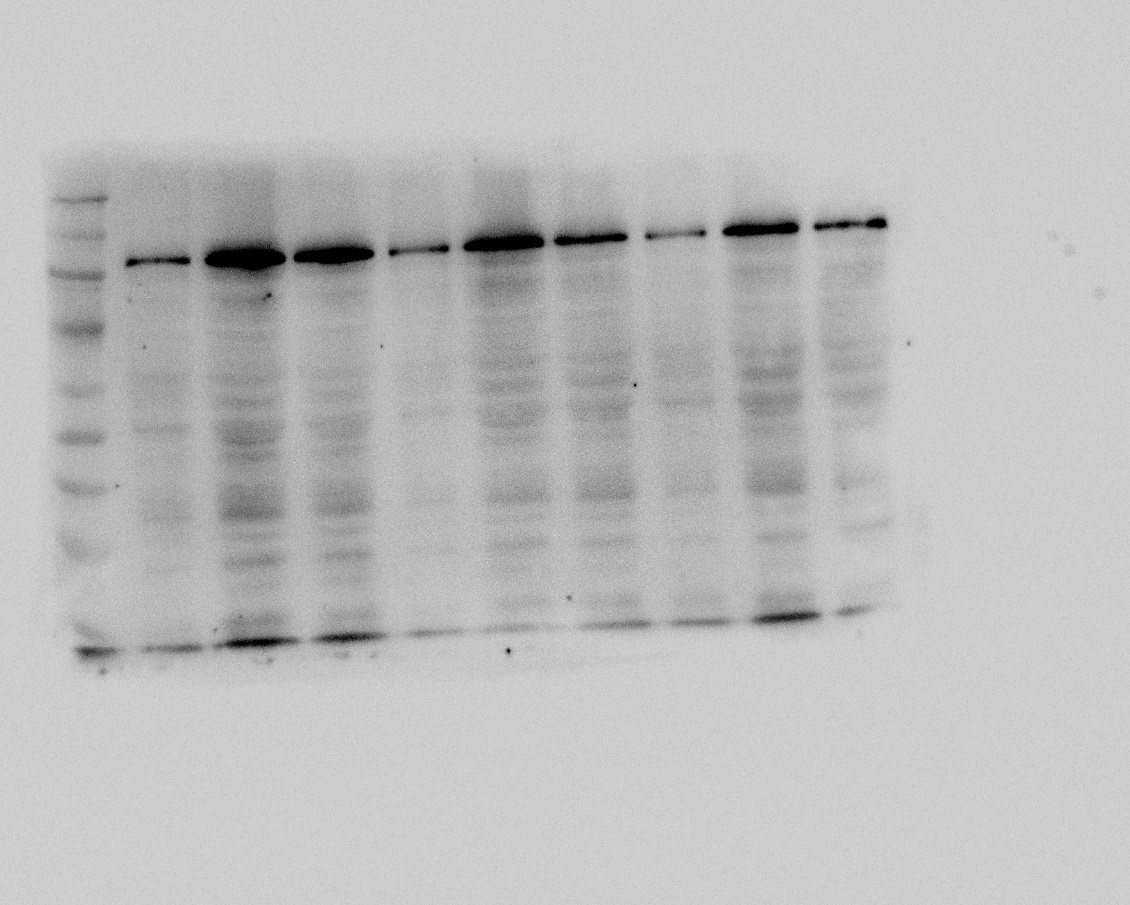


ASC:


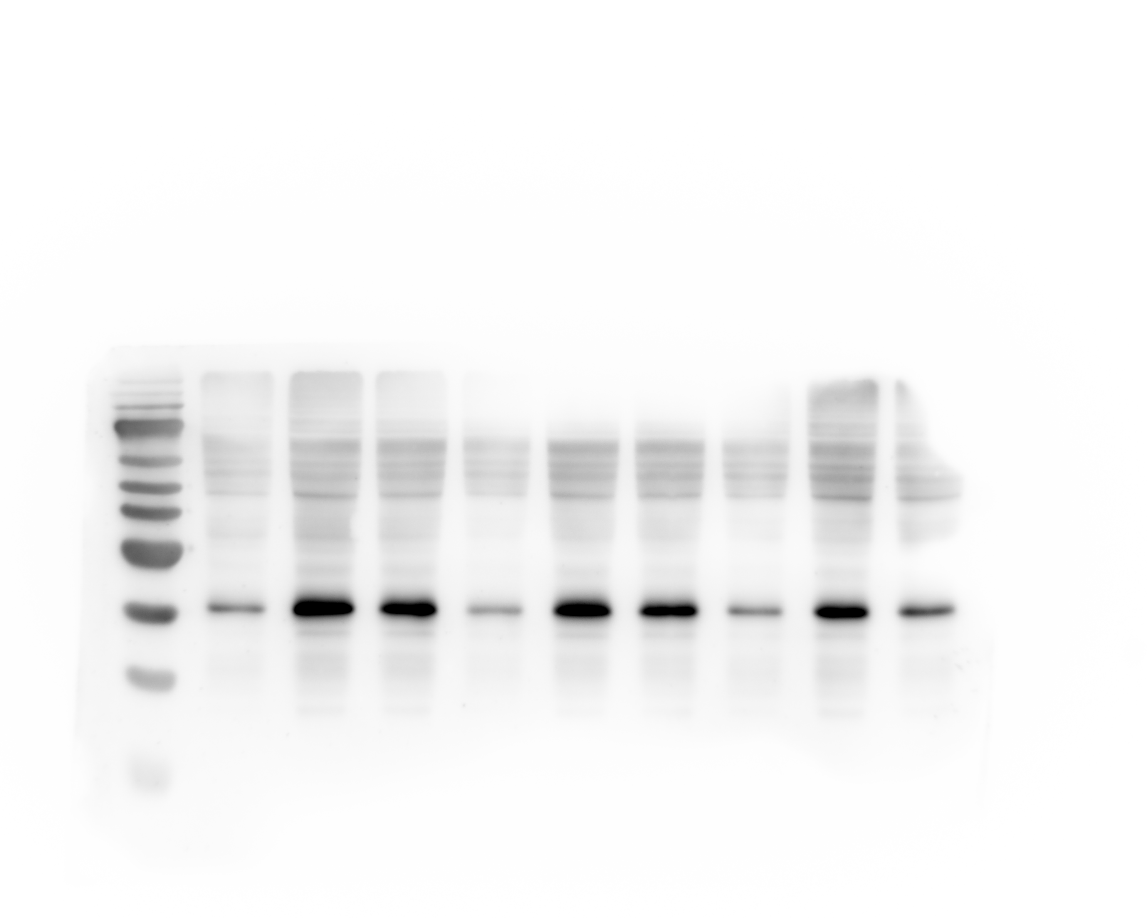


Caspase-1:


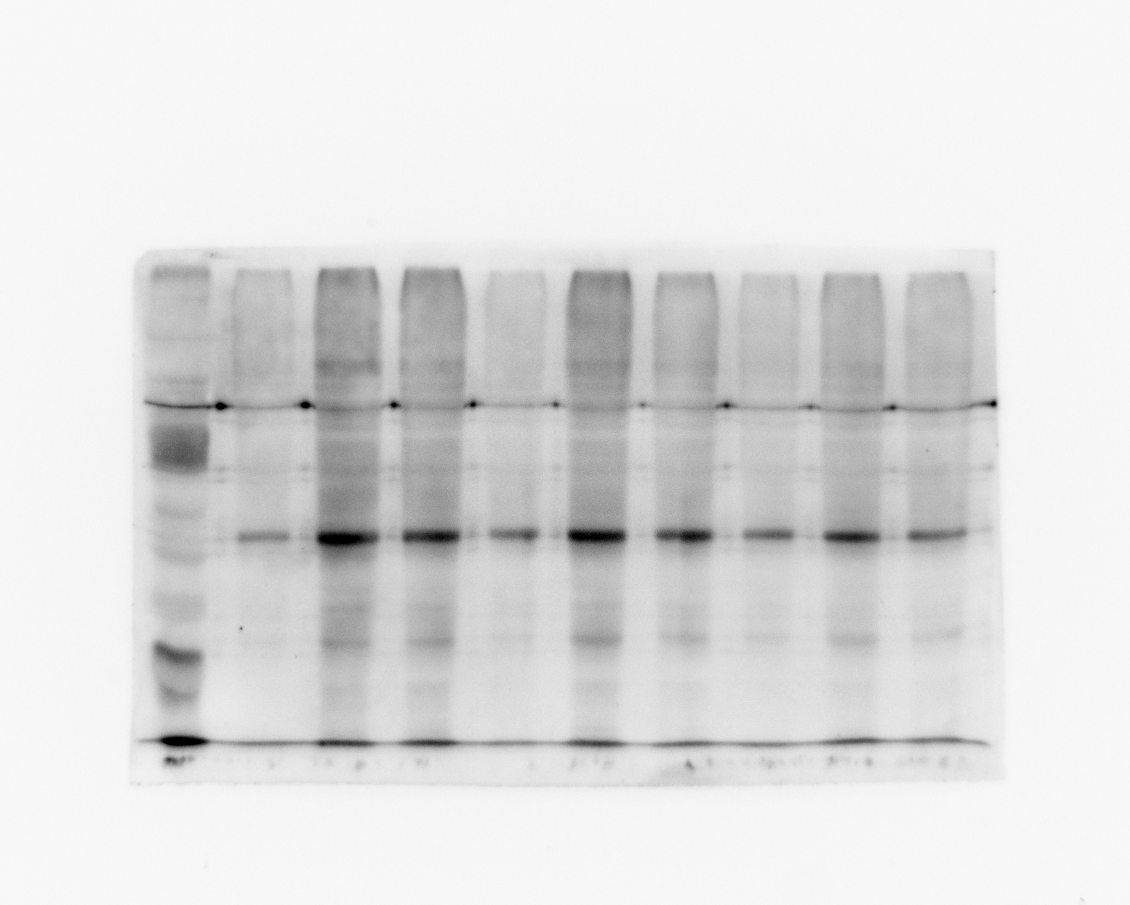


IL-1β：


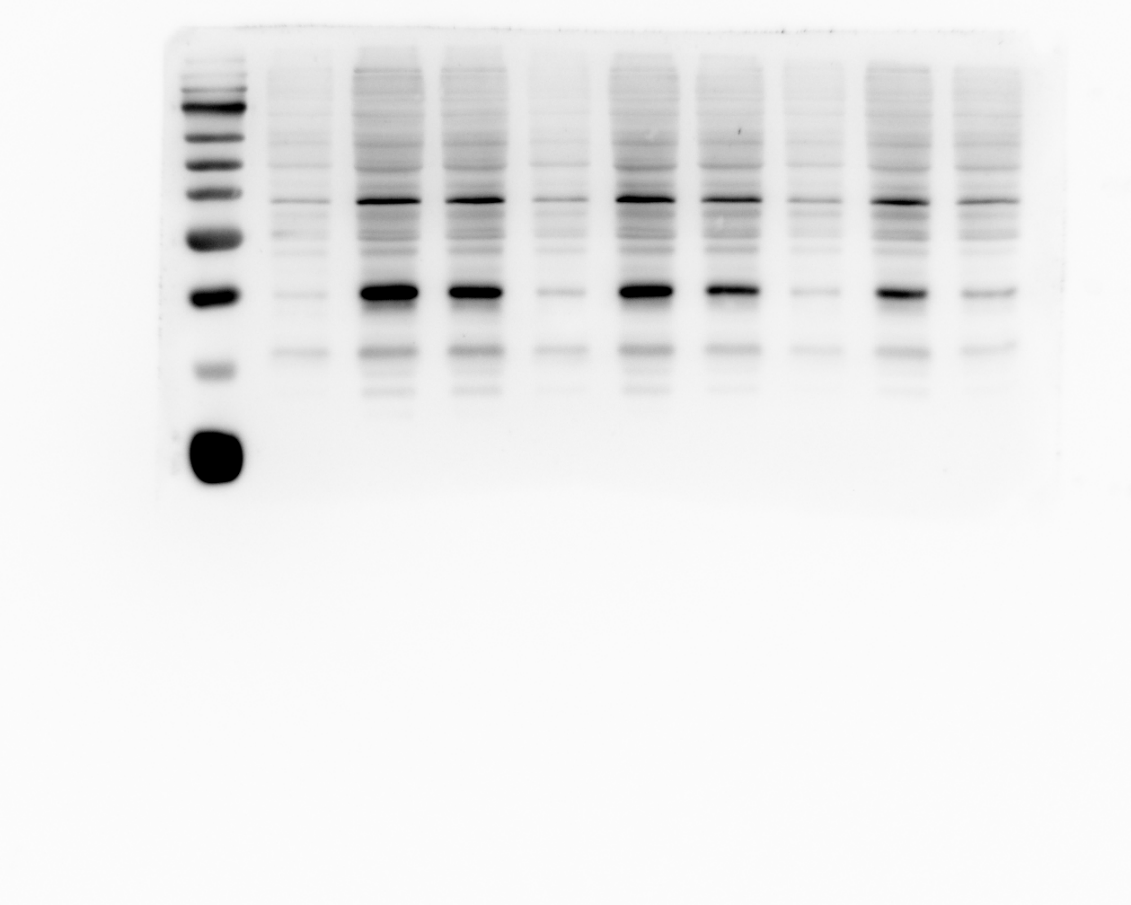


IL-18:


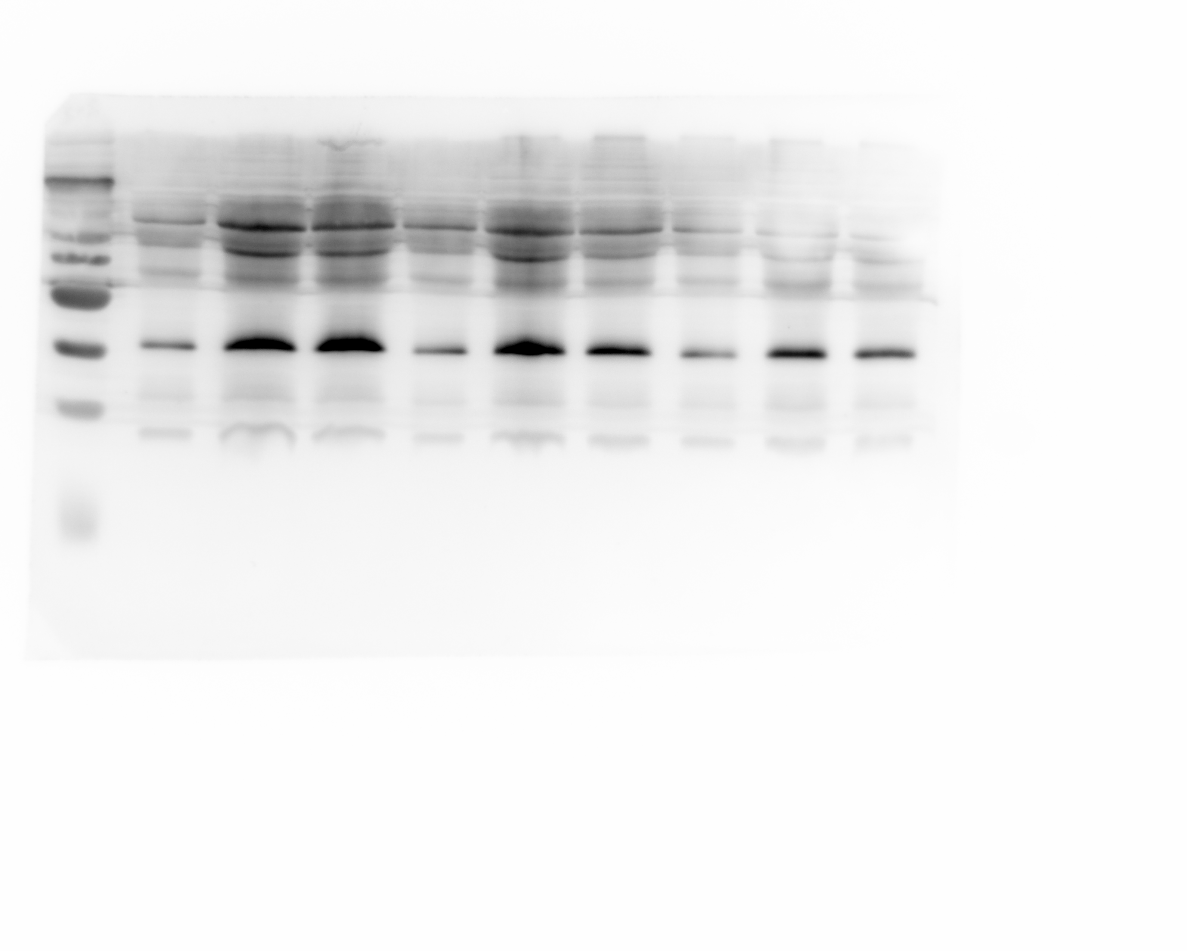


GAPDH:


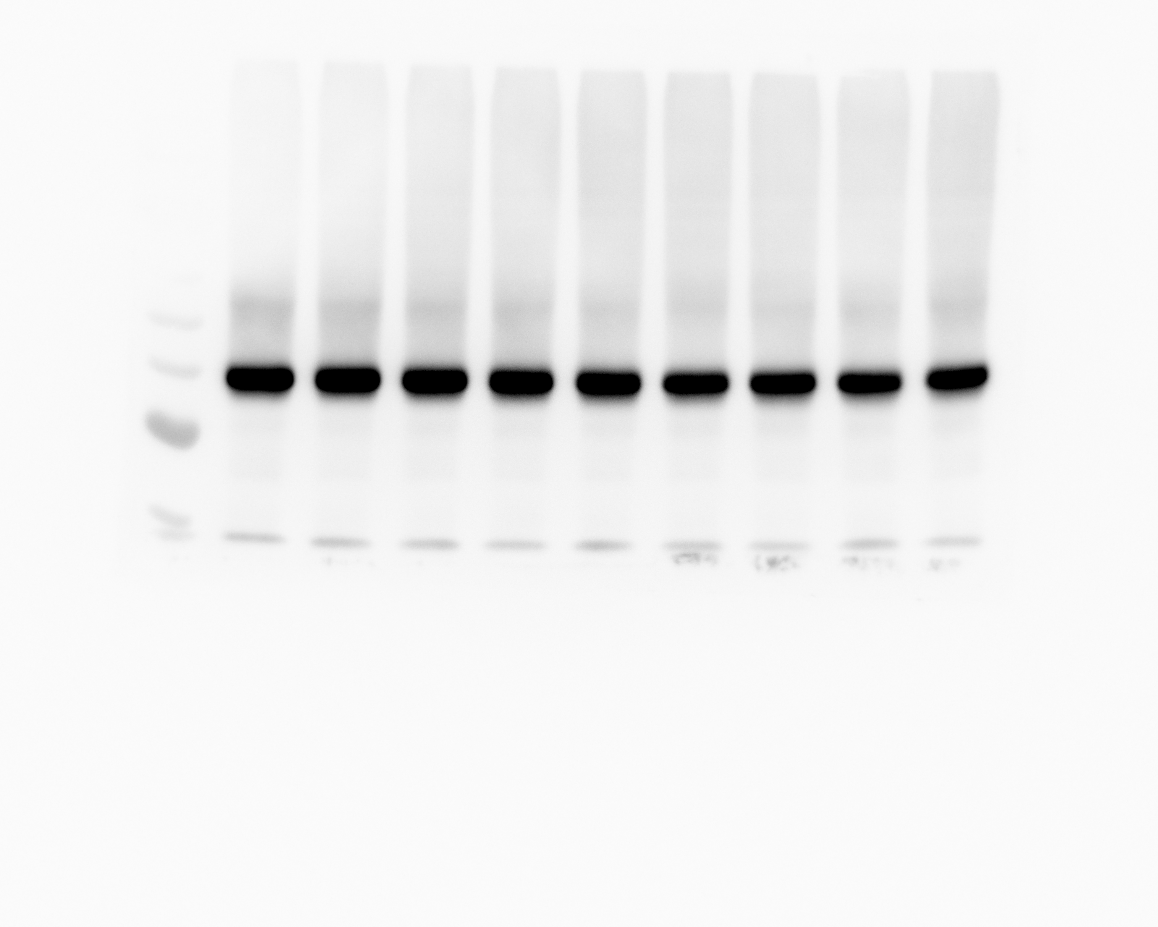

Supplement: Supplementary file 1 — Additional file 1. Original image of Western Blot. [file 12871_2022_1915_MOESM1_ESM.docx]
